# Protective effects of the combination of sodium ferulate and oxymatrine on cecal ligation and puncture-induced sepsis in mice

**DOI:** 10.3892/etm.2014.1604

**Published:** 2014-03-05

**Authors:** MENGXIN XU, WEI WANG, XIAOKUN PEI, SONGMEI SUN, MINGBO XU, ZHIFENG LIU

**Affiliations:** 1 School of Pharmacy, Yantai University, Yantai, Shandong 264005, P.R. China; 2 Beijing SL Pharmaceutical Co., Ltd., Beijing 100049, P.R. China

**Keywords:** sodium ferulate, oxymatrine sepsis, inflammation

## Abstract

The aim of this study was to investigate the effects of the combination of sodium ferulate (SF) and oxymatrine (OMT) on mice with cecal ligation and puncture (CLP)-induced sepsis. Swiss male mice were randomly divided into a control group, CLP group, three SF + OMT groups (3.1+6.9; 6.2+13.8 and 12.3+27.7 mg/kg), SF (6.2 mg/kg) group and OMT (13.8 mg/kg) group. Eight hours after the administration of the drugs, the survival rates and survival times of the animals were monitored. In addition, the lung wet/dry weight (W/D) ratio; alanine aminotransferase (ALT), aspartate aminotransferase (AST) and lactate dehydrogenase (LDH) levels in the serum; the C-reactive protein (CRP), interleukin-6 (IL-6) and interferon-γ (IFN-γ) levels in the serum and lung and liver homogenates; and the malondialdehyde (MDA) and superoxidase dismutase (SOD) levels in the lung and liver homogenates were measured. The bacterial load in the serum was also studied. Following treatment with the combination of SF and OMT, the survival rate increased and the survival time was prolonged; CLP-induced increases in the lung W/D ratio and the levels of ALT, AST, LDH, CRP, IL-6, IFN-γ and MDA were significantly reduced; and the SOD activity levels were increased, compared with those of the untreated animals with CLP-induced sepsis. These results indicated that the combination of SF and OMT induced protective effects against CLP-induced lethal sepsis of mice. The possible mechanism of these effects may be associated with the alleviation of systemic inflammation and diminishment of oxidative injury.

## Introduction

Sepsis, a systemic inflammatory response induced by severe infection, which usually leads to multiple organ dysfunction syndromes, is a common cause of critical illness and mortality in intensive care units (1). Although there have been developments in sophisticated monitoring, antibiotic therapy and glucocorticoid treatment, and advances in the understanding of the molecular underpinnings of sepsis, a number of its complications remain refractory to treatment (2,3). In 2007, the severe sepsis mortality rate was reported to range between 30 and 50%, rising to 80–90% for patients with septic shock and multiple organ failure (4).

In the progression of sepsis, it is considered that the hyperactive systemic inflammatory response, with a large number of inflammatory cytokines and excessive generation of free radicals, is one of the main causes of multiple organ injury. Therefore, accompanying antibiotics treatment, anti-inflammation and anti-oxidation were usually used as important therapeutic strategies for the sepsis. In previous studies, the marked synergetic analgesic and anti-inflammatory effects of the combination of sodium ferulate (SF) and oxymatrine (OMT) have been identified and reported (5–7). Thus, it may be hypothesized that treatment with a combination of SF and OMT will alleviate the inflammatory response and multiple organ injury induced by sepsis.

In the present study, cecal ligation and puncture (CLP)-induced septic mice models were used to evaluate the effects of the combination of SF and OMT based on the anti-inflammatory and antioxidative effects of the treatment. The survival rates and survival times of the animals were monitored. The lung wet/dry weight (W/D) ratio, which represents the degree of lung injury, was calculated. The levels of serum alanine aminotransferase (ALT), aspartate aminotransferase (AST) and lactate dehydrogenase (LDH) were measured, which indicated the degree of injury to the organs. Furthermore, the C-reactive protein (CRP), interleukin-6 (IL-6) and interferon-γ (IFN-γ) levels were assayed, which reflected the anti-inflammatory efficacy of the treatment. Also, in order to investigate the oxidative injury, the levels of malondialdehyde (MDA), a biomarker of oxidative injury, and superoxidase dismutase (SOD), an important free radical scavenger *in vivo*, were measured.

## Materials and methods

### Drugs and chemicals

SF [molecular formula: C_10_H_9_NaO_4_.2H_2_O; molecular weight: 252.20; CAS: 24276-84-4; high-performance liquid chromatography (HPLC) purity: >99%] and OMT (molecular formula: C_15_H_24_N_2_O_2_.H_2_O; molecular weight: 282.38; CAS: 16837-52-8; HPLC purity: >98%) were provided by Beijing SL Pharmaceutical Co., Ltd. (Beijing, China). The optimal ratio (molar ratio = 1:2) of the combination of SF and OMT was obtained by pharmaceutical and pharmacological tests. When the molar ratio of SF and OMT was 1:2, the solution system was the most stable with a pH value of 7.0, and the pharmacological activity was also strongest (unpublished data).

### Animals

Swiss male mice (18–22 g; Shandong Luye Pharmaceutical Co., Ltd, Yantai, China; Quality Certificated Number: Lu 20090013) were used. The animals were maintained under standard conditions (12-h light/dark cycle, temperature: 23±2°C, humidity: 55±5%) for 3–7 days for acclimatization to the surrounding environment. The animals had access to food and water *ad libitum*. Within the 12 h prior to the experiment, only water was supplied. In accordance with the National Institutes of Health Guide for the Care and Use of Laboratory Animals (Eighth edition, 2012), all procedures conducted in these experiments were approved by the Experimental Animal Management Center of Yantai University (Yantai, China).

### Model of CLP-induced sepsis

The animals were randomly divided into seven groups with 30 mice in each group. The groups were as follows: i) Control (saline); ii) CLP (saline); iii) SF + OMT (3.1+6.9 mg/kg); iv) SF + OMT (6.2+13.8 mg/kg); v) SF + OMT (12.3+27.7 mg/kg); vi) SF (6.2 mg/kg); and vii) OMT (13.8 mg/kg). Following anesthetization with chloraldurate (3%), the animals underwent surgery with reference to the methods of Baker *et al* (8). Briefly, a midline incision was made below the diaphragm to expose the cecum. The cecum was ligated immediately below the ileocecal valve with 1-0 silk so that intestinal continuity was maintained. Following two punctures with a five-gauge needle, the cecum was gently compressed until fecal matter was extruded. Subsequently, the cecum was gently returned to the abdomen, and the incision was closed in layers with a 2-0 silk ligature suture. The animals in the control group underwent a laparotomy, and the cecum was manipulated, but not ligated and perforated. At the end of the surgery, the corresponding drugs or saline were administered intraperitoneally; the quantity of saline administered was 20 ml/kg body weight. Following resuscitation of the animals, food and water were provided *ad libitum*.

### Survival rate and survival time

In each group, the survival rates and survival times of 10 mice were monitored. Following the CLP surgery, the animals were carefully observed for ~8 h, followed by observation every 8 h for 24 h. The time of mortality was recorded. If an animal succumbed between the observations at 8 and 16 h, the survival time was recorded as 16 h, and if the animal had not succumbed by the 24 h point, the survival time was recorded as 24 h. After 24 h, the surviving mice were sacrificed with carbon dioxide anesthesia.

### Bacterial load determination

In a preliminary experiment, the animals began to succumb at ~10 h after CLP surgery. Thus in the present study, 8 h after the CLP surgery, 10 mice from each group were randomly selected and blood was obtained sterilely by percutaneous cardiac puncture, then diluted 100-fold with phosphate-buffered saline (PBS). The bacterial load was determined with reference to the methods of Standage *et al* (9). Briefly, 200 μl diluted blood from each mouse was plated on a chocolate agar plate (Thermo Fisher Scientific Inc., Pittsburgh, PA, USA). The plates were incubated for 24 h at 37°C and the number of colony forming units (CFUs) was counted.

### Lung W/D ratio calculation

Following the collection of the blood for bacterial load determination, the animals were sacrificed and the lungs were excised immediately. The lungs of each animal (n=10) were weighed, and then dried in an oven at 70°C for 48 h and re-weighed. The W/D ratio was calculated using the following formula: W/D ratio = wet weight / dry weight.

### Separation of serum and preparation of lung and liver homogenates

Blood was collected from an eyeball of each of the 10 mice remaining in each group following anesthesia with diethyl ether. The serum was separated by centrifugation at 600 × g for 10 min and stored at −80°C for further biochemical analysis. Subsequently, the animals were sacrificed. The lungs and livers were excised and homogenized in PBS on ice to prepare a 10% homogenate using a Vertishear tissue homogenizer (Virtis, Gardiner, NY, USA). The homogenate was also stored at −80°C for further biochemical analysis.

### Biochemical analysis

The levels of ALT, AST and LDH in the serum were measured by routine laboratory methods using a Toshiba Automatic analyzer (TOSHIBA TBA-40FR ACCUTE, Toshiba Corporation, Tokyo, Japan). The levels of CRP, IL-6 and IFN-γ in the serum and in the lung and liver homogenates were measured by enzyme-linked immunosorbent assay (ELISA) kits according to the manufacturer’s instructions. The ELISA kits for the determination of the levels of CRP, IFN-γ and IL-6 were produced by Groundwork Biotechnology Diagnosticate Ltd (San Diego, CA, USA). The MDA content and SOD activity levels in the lung and liver homogenates were measured as described previously (10,11). Briefly, the MDA content was detected by the thiobarbituric acid method with a maximal absorbance at 532 nm, and the SOD activity levels were measured based on the SOD-mediated inhibition of nitrite formation from hydroxyammonium in the presence of O_2_^•−^ generators (xanthine/xanthine oxidase) (10). The MDA and SOD test kits were produced by Nanjing Jiancheng Bioengineering Institute (Nanjing, China), and have been used in numerous studies (12,13).

### Statistical analysis

All data are presented as the mean ± standard error of the mean and were analyzed by one-way analysis of variance, with Statistical Product and Service Solutions software, version 17.0 (SPSS, Inc., Chicago, IL, USA). The χ^2^ test was used to compare the differences of the survival rates between two groups. P<0.05 was considered to indicate a statistically significant difference.

## Results

### Effects of SF and OMT used in combination or alone on the survival rate and survival time

**Figure 1:**
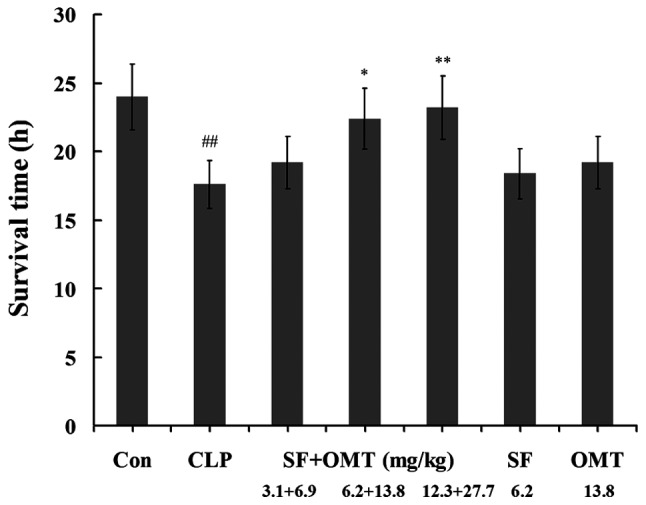
Effects of the combination of SF and OMT on the survival time of CLP-induced septic mice. Con, CLP, SF and OMT represent the control group, CLP group, SF (6.2 mg/kg) and OMT (13.8 mg/kg) groups, respectively. SF + OMT represents the SF and OMT combination groups. The data are expressed as the mean ± standard error of the mean. n=10 in each group. ^##^P<0.01, versus the control group. ^*^P<0.05 and ^**^P<0.01, versus the CLP group. CLP, cecal ligation and puncture; SF, sodium ferulate; OMT, oxymatrine.

As shown in Table I, within 24 h after the surgery, all animals survived in the control group. In the CLP group, the animal survival rate was 20% at 16 h and all animals had died by 24 h. Treatment with the combination of SF and OMT at the medium and high doses significantly increased the survival rate and prolonged the survival time compared with those of the CLP group (Fig. 1). At 24 h, the survival rates were 20, 40 and 50% in the SF + OMT 3.1+6.9; 6.2+13.8; and 12.3+27.7 mg/kg combination groups, respectively. Treatment with either SF (6.2 mg/kg) or OMT (13.8 mg/kg) alone did not significantly increase the survival rate at 24 h after the surgery (0 and 10%, respectively), or prolong the survival time compared with those of the combination groups.

### Effects of SF and OMT used in combination or alone on the lung W/D ratio

**Figure 2:**
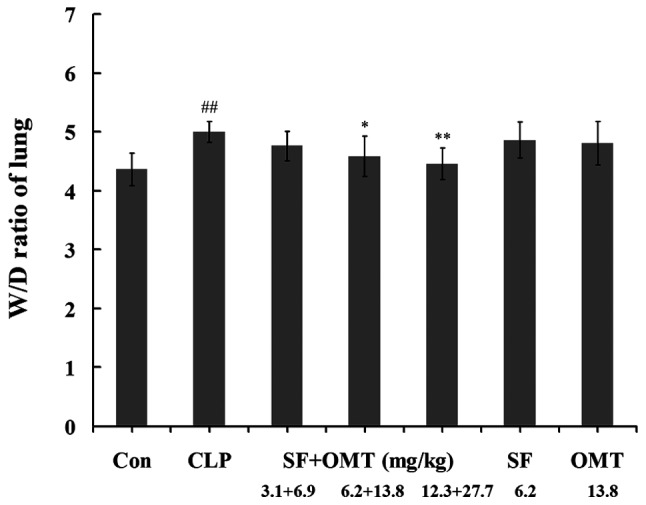
Effects of the combination of SF and OMT on the lung W/D ratio of CLP-induced septic mice. Con, CLP, SF and OMT represent the control group, CLP group, SF (6.2 mg/kg) and OMT (13.8 mg/kg) groups, respectively. SF + OMT represents the SF and OMT combination groups. The lung W/D ratio was determined at 8 h after the CLP surgery. The data are expressed as the mean ± standard error of the mean. ^##^P<0.01, versus the control group. ^*^P<0.05 and ^**^P<0.01, versus the CLP group. W/D, wet/dry weight; CLP, cecal ligation and puncture; SF, sodium ferulate; OMT, oxymatrine.

As shown in Fig. 2, the lung W/D ratio increased significantly in the CLP group compared with that in the control group. The combination treatment reduced the lung W/D ratio compared with that of the CLP group (P<0.05 in the medium dose group; P<0.01 in the high dose group). No significant efficacy was observed in the groups treated with SF (6.2 mg/kg) or OMT (13.8 mg/kg) alone.

### Effects of SF and OMT used in combination or alone on the levels of ALT, AST and LDH in serum

**Figure 3:**
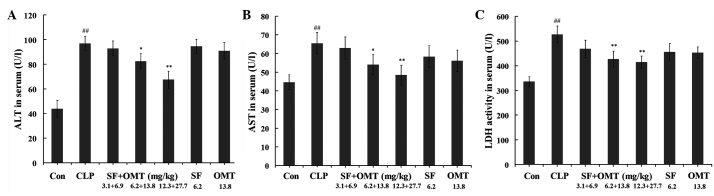
Effects of the combination of SF and OMT on the levels of (A) ALT, (B) AST and (C) LDH in the serum of CLP-induced septic mice. Con, CLP, SF and OMT represent the control group, CLP group, SF (6.2 mg/kg) and OMT (13.8 mg/kg) groups, respectively. SF + OMT represents the SF and OMT combination groups. The data are expressed as the mean ± standard error of the mean. n=10 in each group. ^##^P<0.01, versus the control group. ^*^P<0.05 and ^**^P<0.01, versus the CLP group. ALT, alanine aminotransferase; CLP, cecal ligation and puncture; SF, sodium ferulate; OMT, oxymatrine; AST, aspartate aminotransferase; LDH, lactate dehydrogenase.

As shown in Fig. 3, following the CLP surgery, the serum ALT, AST and LDH levels notably increased in the CLP group compared with those in the control group. In the medium and high dose groups of the SF and OMT combination treatment (SF + OMT 6.2+13.8 and 12.3+27.7 mg/kg) the serum LDH, ALT and AST levels significantly decreased compared with those in the CLP group. No significant inhibitory effects on serum LDH, ALT and AST levels were observed in the groups treated with SF (6.2 mg/kg) or OMT (13.8 mg/kg) alone.

### Effects of SF and OMT used in combination or alone on the levels of CRP, IL-6 and IFN-γ in serum

**Figure 4:**
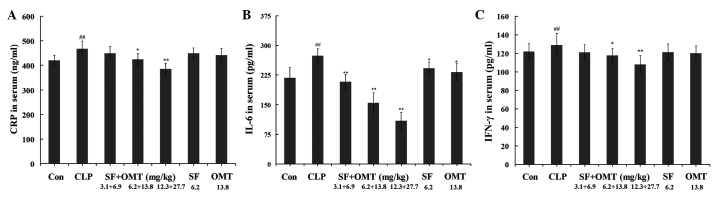
Effects of the combination of SF and OMT on the levels of (A) CRP, (B) IL-6 and (C) IFN-γ in the serum of CLP-induced septic mice. Con, CLP, SF and OMT represent the control group, CLP group, SF (6.2 mg/kg) and OMT (13.8 mg/kg) groups, respectively. SF + OMT represents the SF and OMT combination groups. The data are expressed as the mean ± standard error of the mean. n=10 in each group. ^##^P<0.01, versus the control group. ^*^P<0.05 and ^**^P<0.01, versus the CLP group. CRP, C-reactive protein; CLP, cecal ligation and puncture; SF, sodium ferulate; OMT, oxymatrine; IL-6, interleukin-6; IFN-γ, interferon-γ.

Following the CLP surgery (8 h), the serum CRP, IL-6 and IFN-γ levels all significantly increased in the CLP group compared with those in the control group (Fig. 4). In all combination treatment groups (SF + OMT 3.1+6.9, 6.2+13.8 and 12.3+27.7 mg/kg), the levels of IL-6 in the serum were significantly reduced in a dose-dependent manner compared with those in the CLP group (P<0.01). The levels of CRP and IFN-γ in the serum were significantly decreased in the high and medium dose groups of the combination treatment compared with those in the CLP group (P<0.05 in the medium dose group; P<0.01 in the high dose group). With the exception of the levels of IL-6 in the serum, which decreased significantly (P<0.05) in the SF (6.2 mg/kg) and OMT (13.8 mg/kg) alone groups, the other measured indices did not exhibit significant changes in the SF or OMT alone treatment groups compared with the levels in the CLP group.

### Effects of SF and OMT used in combination or alone on the levels of CRP, IL-6 and IFN-γ in the lung homogenate

**Figure 5:**
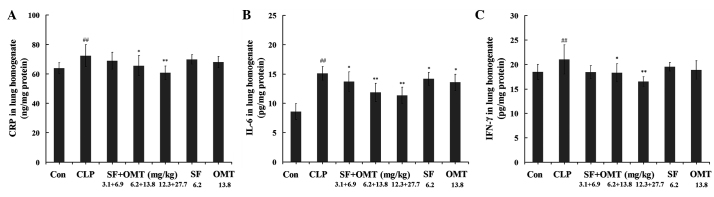
Effects of the combination of SF and OMT on the levels of (A) CRP, (B) IL-6 and (C) IFN-γ in the lung homogenates of CLP-induced septic mice. Con, CLP, SF and OMT represent the control group, CLP group, SF (6.2 mg/kg) and OMT (13.8 mg/kg) groups, respectively. SF + OMT represents the SF and OMT combination groups. The data are expressed as the mean ± standard error of the mean. n=10 in each group. ^##^P<0.01, versus the control group. ^*^P<0.05 and ^**^P<0.01, versus the CLP group. CRP, C-reactive protein; CLP, cecal ligation and puncture; SF, sodium ferulate; OMT, oxymatrine; IL-6, interleukin-6; IFN-γ, interferon-γ.

As shown in Fig. 5, following the CLP surgery, the levels of CRP, IL-6 and IFN-γ in lung homogenate notably increased in the CLP group compared with those in the control group. Treatment with the combination of SF and OMT (SF + OMT 3.1+6.9, 6.2+13.8 and 12.3+27.7 mg/kg) significantly reduced the levels of IL-6 compared with those in the CLP group (P<0.05 in the low dose group; P<0.01 in the high and medium dose groups). The CRP and IFN-γ levels in the lung homogenate were significantly reduced in the high and medium dose groups of the combination treatment compared with those in the CLP group (P<0.05 in the medium dose group; P<0.01 in the high dose group). With the exception of the levels of IL-6 in the lung homogenate, which decreased significantly in the SF (6.2 mg/kg) and OMT (13.8 mg/kg) groups (P<0.05), the other measured indices did not exhibit significant changes in the SF or OMT alone treatment groups compared with the levels in the CLP group.

### Effects of SF and OMT used in combination or alone on the levels of CRP, IL-6 and IFN-γ in the liver homogenate

**Figure 6:**
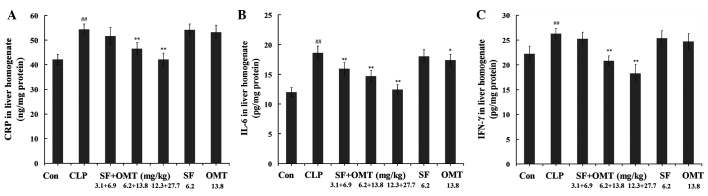
Effects of the combination of SF and OMT on the levels of (A) CRP, (B) IL-6 and (C) IFN-γ in the liver homogenates of CLP-induced septic mice. Con, CLP, SF and OMT represent the control group, CLP group, SF (6.2 mg/kg) and OMT (13.8 mg/kg) groups, respectively. SF + OMT represents the SF and OMT combination groups. The data are expressed as the mean ± standard error of the mean. n=10 in each group. ^##^P<0.01, versus the control group. ^*^P<0.05 and ^**^P<0.01, versus the CLP group. CRP, C-reactive protein; CLP, cecal ligation and puncture; SF, sodium ferulate; OMT, oxymatrine; IL-6, interleukin-6; IFN-γ, interferon-γ.

As shown in Fig. 6, following the CLP surgery, the levels of CRP, IL-6 and IFN-γ in the liver homogenate notably increased in the CLP group compared with those in the control group. Treatment with the combination of SF and OMT (SF + OMT 3.1+6.9, 6.2+13.8 and 12.3+27.7 mg/kg) significantly reduced the levels of IL-6 compared with those in the CLP group (P<0.01). The CRP and IFN-γ levels in the liver homogenate were significantly decreased in the high and medium dose groups of the combination treatment compared with those in the CLP group (P<0.01). With the exception of the levels of IL-6 in the liver homogenate, which reduced significantly (P<0.05) in the OMT (13.8 mg/kg) group, the other measured indices did not exhibit significant changes in the SF or OMT alone treatment groups compared with the levels in the CLP group.

### Effects of SF and OMT used in combination or alone on the levels of MDA and SOD activity in the lung homogenate

**Figure 7:**
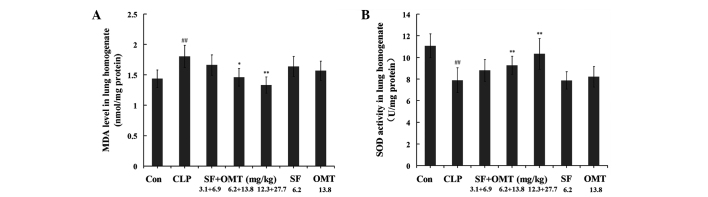
Effects of the combination of SF and OMT on the levels of (A) MDA and (B) SOD activity in the lung homogenates of CLP-induced septic mice. Con, CLP, SF and OMT represent the control group, CLP group, SF (6.2 mg/kg) and OMT (13.8 mg/kg) groups, respectively. SF + OMT represents the SF and OMT combination groups. The data are expressed as the mean ± standard error of the mean. n=10 in each group. ^##^P<0.01, versus the control group. ^*^P<0.05 and ^**^P<0.01, versus the CLP group. MDA, malondialdehyde; CLP, cecal ligation and puncture; SF, sodium ferulate; OMT, oxymatrine; SOD, superoxidase dismutase.

As shown in Fig. 7, the CLP surgery resulted in a marked increase in the MDA levels and reduction in the SOD activity levels in the lung homogenates of the CLP group compared with those in the control group. Compared with those in the CLP group, in the combination treatment groups the MDA levels significantly decreased (P<0.05 in the medium dose group; P<0.01 in the high dose group) and the SOD activity levels increased (P<0.01 in the medium and high dose groups) in the lung homogenate. In the groups treated with SF (6.2 mg/kg) or OMT (13.8 mg/kg) alone, the MDA and SOD activity levels in the lung homogenate did not exhibit significant changes compared with those in the CLP group.

### Effects of SF and OMT used in combination or alone on the levels of MDA and SOD activity in the liver homogenates

**Figure 8:**
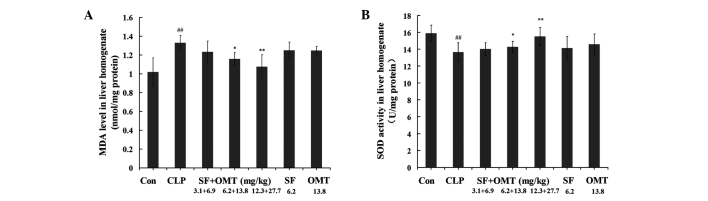
Effects of the combination of SF and OMT on the levels of (A) MDA and (B) SOD activity in the liver homogenates of CLP-induced septic mice. Con, CLP, SF and OMT represent the control group, CLP group, SF (6.2 mg/kg) and OMT (13.8 mg/kg) groups, respectively. SF + OMT represents the SF and OMT combination groups. The data are expressed as the mean ± standard error of the mean. n=10 in each group. ^##^P<0.01, versus the control group. ^*^P<0.05 and ^**^P<0.01, versus the CLP group. MDA, malondialdehyde; CLP, cecal ligation and puncture; SF, sodium ferulate; OMT, oxymatrine; SOD, superoxidase dismutase.

As shown in Fig. 8, the CLP surgery resulted in a marked increase of the MDA levels and reduction in the SOD activity levels in the liver homogenates in the CLP group compared with those in the control group. Compared with those of the CLP group, in the combination treatment groups the MDA levels significantly decreased and the SOD activity levels increased in a dose-dependent manner in the liver homogenate. In the group treated with SF (6.2 mg/kg) or OMT (13.8 mg/kg) alone, the MDA and SOD activity levels in the liver homogenate did not exhibit significant differences compared with those in the CLP group.

### Effects of the combination of SF and OMT on the bacterial load in the blood

After the CLP surgery (8 h), the bacterial load of the blood was markedly increased compared with that in the control group. Compared with that in the CLP group, no significant difference in number of CFUs was detected in the combination treatment groups (SF + OMT 3.1+6.9, 6.2+13.8 and 12.3+27.7 mg/kg), or SF (6.2 mg/kg) and OMT (13.8 mg/kg) groups (P>0.05; data not shown).

## Discussion

SF, one of the active ingredients isolated from the Chinese herb Radix *Angelica sinensis* (Oliv.) Diels, exerts antioxidative, anti-inflammatory and platelet aggregation inhibitory effects, and free radical-scavenging activities (14,15). SF has been approved by the State Food and Drugs Administration of China (Beijing, China) as a drug for the clinical treatment of cardiovascular and cerebrovascular diseases. OMT, a component of the Chinese herb Radix *Sophora flavescent* Ait., has been widely used for the treatment of chronic hepatitis in China, and its positive pharmacological effects on the regulation of the immune reaction, reduction of hypersensitive reactions and inhibition of histamine release have been demonstrated by *in vitro* and *in vivo* studies (16,17). Previous studies have shown that the combination of SF and OMT has a synergistic anti-inflammatory effect and may protect against lipopolysaccharide (LPS)-induced lung injury (7). Therefore, the present study used the CLP-induced model of sepsis to further corroborate the anti-inflammatory effect of the combination of SF and OMT and aimed to demonstrate its protective effect on sepsis-induced organ injury.

Sepsis resulting from CLP in animals is an accepted animal model that closely imitates the physiological changes observed during the progression of sepsis in humans (18). This model has been shown to accurately reproduce the sepsis sequelae with regard to the hyperactive inflammatory process, generation of cytokines and development of multiorgan failure that leads to mortality (19). Control of the extent of damage resulting from cecum perforation may effectively control the survival rate and survival time of mice. In the present study, none of the animals died within 8 h after the CLP surgery, and of the animals in the CLP group, 20% survived to the 16 h point and none survived to the 24 h point. These results indicated that CLP-induced sepsis was a suitable model to duplicate the pathological process of acute inflammatory reactions and validate the anti-inflammatory effect of the SF and OMT combination drug treatment. Treatment with the combination of SF and OMT at the medium and high doses significantly increased the survival rate and prolonged the survival time of the mice with CLP-induced sepsis effectively compared with those of the CLP-induced septic models without treatment. These results supported our hypothesis concerning the protective effect of the SF and OMT combination treatment on CLP-induced sepsis.

Sepsis-induced multiple organ injury is the main cause of septic shock and patient mortality, in which the lung is the primary target organ and importantly, mortality is mainly caused by irreversible lung injury, termed *‘*shock lung’ (20). The water content of the lung tissue, which represents the degree of lung injury, is reflected by the lung W/D ratio. The serum ALT, AST and LDH levels are biochemical markers for the extent of injury to the liver and other organs and are easily measured using a routine automatic analyzer. The increased levels of ALT, AST and LDH in serum indicate the degree of organ injury (21,22). Thus, following treatment with the combination of SF and OMT in the present study, the notable reduction in the levels of ALT, AST and LDH suggested that the cellular injury to the liver and other organs induced by CLP was attenuated. These results exhibited the protective effect of the combination of SF and OMT on CLP-induced organ injury.

For modulation of a number of healing processes, the important inflammatory mediators, including CRP, IL-6 and IFN-γ, are rapidly induced in the early stage of the inflammatory response. However, if overproduced, these mediators may exacerbate the severity of multiple inflammatory diseases, particularly in sepsis (23–26).

CRP is a type of reactive protein in the acute inflammatory phase (27) and shows a significant positive correlation with the extent of tissue injury. As an indicator of the systemic inflammatory reaction, CRP may exhibit proinflammatory effects by activating the complement system and inducing the production of inflammatory cytokines and tissue factor in monocytes (27,28). CRP is a marker of inflammatory reaction and predicts the risk of the occurrence of organ injury (29). IL-6 is an early inflammatory mediator that is markedly upregulated in the serum of patients with sepsis (30,31). As a proinflammatory cytokine (32), IL-6 has numerous biological features and plays an important role in the process of inflammation (33). IFN-γ is a potent proinflammatory cytokine and contributes to innate immunity. IFN-γ is a risk factor for the occurrence of organ injury (34). A previous study showed that following treatment with the combination of SF and OMT, the CRP, IL-6 and IFN-γ genes were all synergistically downregulated in LPS-stimulated RAW 264.7 cells (5). In order to confirm that the anti-inflammatory effect of the combination of SF and OMT was associated with the inflammatory cytokines, 10 mice in each group of the present study were sacrificed and the levels of CRP, IL-6 and IFN-γ in serum, lung and liver homogenate were detected at 8 h after the CLP surgery, at which point none of the animals had died. It was detected that the levels of the inflammatory cytokines had all increased markedly compared with those in the control group. As the results showed, treatment with the combination of SF and OMT significantly reduced the increased CRP, IL-6 and IFN-γ levels induced by CLP in the serum, lung and liver homogenates. These results were consistent with the reverse transcription-polymerase chain reaction results published in a previous study (5) and further confirmed the anti-inflammatory effect of the combination of SF and OMT involved in the protective mechanism of the organs.

Sepsis is frequently associated with the generation of a large number of free radicals and an excessive amount of free radicals causes oxidative tissue injury, which is considered to trigger the inflammatory response and contribute to organ injury (35,36). One of histopathological causes of sepsis is the overproduction of reactive oxygen species. MDA is produced by lipid peroxidation inside cells. The MDA content of an organism reflects the degree of lipid peroxidation (37). The MDA levels are a valuable biomarker of oxidative injury in tissues and indirectly present the degree of cellular injury (38,39). SOD is an antioxidant enzyme that removes superoxide free radicals and protects against cellular injury from free radicals (40). The enhancement of the oxidation reaction is reflected by a reduction in the levels of SOD activity. In the present study, the MDA levels in the lung and liver tissue were markedly increased and the SOD activity levels were markedly reduced by CLP, compared with those in the control group. However, the increased MDA levels were significantly reduced and the decreased SOD activity levels were significantly increased by the medium and high doses of the combination SF and OMT treatment. Thus, the results indicate that the combination treatment alleviates the extent of the oxidative injury in the lung and liver tissues. These results were in accordance with those of a previous study (7), and indicated that the combination treatment significantly attenuates the LPS-induced reduction in the levels of SOD activity.

These observations suggest that the bacterial load may increase due to the anti-inflammatory effect of SF and OMT. Thus, in the present study the bacterial load in mice of all groups was detected. No significant differences were observed between the bacterial load of the CLP group and those of the groups treated with a combination of SF and OMT or the drugs alone at 8 h after the CLP surgery. This suggested that treatment with the combination of SF and OMT did not increase the bacterial load in the early stage of the inflammatory response. In an *in vitro* experiment conducted by authors of the present study, it was demonstrated that following treatment with the combination of SF and OMT, neither an antibiotic effect on certain Gram-positive and Gram-negative bacteria, nor an effect on the antibiotic effect of penicillin and streptomycin (data not shown) were observed.

The elucidation of the anti-inflammatory effect of the combination of SF and OMT is likely to provide potential therapeutic approaches, for example the combination treatment may be a substitute for the corticosteroid drugs used to treat sepsis. To achieve this goal, further studies should be conducted, with the aim of exploring whether the combination of SF and OMT treatment influences the defense system of the body and its superior advantage compared with corticosteroid drugs. The present study only suggested that an anti-inflammatory strategy had a significant therapeutic effect in the early stage of a serious inflammatory disease, and that alleviation of the inflammatory response is likely to be beneficial for organ protection, as it would provide the time for recovery of patients. Beyond all question, antibiotic therapy should be the first-line strategy for the treatment of serious infectious disease.

In order to further test the synergistic anti-inflammatory effect of the combination of SF and OMT in the present study, SF and OMT treatment alone groups were designed as further control groups. When used in the same doses as those in the medium dose combination treatment group, no clear anti-inflammatory or antioxidative effects were observed in the mice treated with SF or OMT alone, with the exception of a slight improvement in the IL-6 levels. These results indirectly provided more data further confirming the synergistic effect of the SF and OMT combination treatment. Detailed data analyzing the synergistic effect of the combination of SF and OMT have been published in a previous study (5,7). Also, data regarding the optimization of the dose ratio of SF and OMT may be reported by the authors of the present study in the future.

In conclusion, the combination of SF and OMT had protective effects against CLP-induced sepsis in mice. The possible mechanisms of these effects may be associated with the alleviation of systemic inflammation, including reductions in IL-6, CRP and IFN-γ levels, and the diminishment of oxidative injury.

## Figures and Tables

**Table I:** Effects of the combination of SF and OMT on the survival rate of CLP-induced septic mice (n=10 per group).

		Survival rate (%)	
		
Group	Dose (mg/kg)	16 h	24 h
Con	-	100	100
CLP	-	20^a^	0^a^
SF+OMT (low dose)	3.1+6.9	40	20
SF+OMT (medium dose)	6.2+13.8	80^a^	40^b^
SF+OMT (high dose)	12.3+27.7	90^c^	50^c^
SF	6.2	30	0
OMT	13.8	40	10

Con, CLP, SF and OMT represent the control group, CLP group and SF and OMT groups, respectively. SF + OMT represents the SF and OMT combination groups. The statistical difference between two groups was analyzed by χ^2^ test.

a P<0.01, versus the control group.

b P<0.05 and

c P<0.01, versus the CLP group.

SF, sodium ferulate; OMT, oxymatrine; CLP, cecal ligation and puncture.
